# Tools for assessing the methodological limitations of a QES—a short note

**DOI:** 10.1186/s13643-024-02511-6

**Published:** 2024-04-06

**Authors:** Heid Nøkleby, Heather Melanie R. Ames, Lars Jørun Langøien, Christine Hillestad Hestevik

**Affiliations:** Norwegian Institute of Public Health, Oslo, Norway

**Keywords:** Qualitative evidence synthesis, QES, Assessment tool, Methodological limitations

## Abstract

The increasing prevalence and application of qualitative evidence syntheses (QES) in decision-making processes underscore the need for robust tools to assess the methodological limitations of a completed QES. This commentary discusses the limitations of three existing tools and presents the authors’ efforts to address this gap. Through a simple comparative analysis, the three tools are examined in terms of their coverage of essential topic areas. The examination finds that existing assessment tools lack comprehensive coverage, clarity, and grounding in qualitative research principles. The authors advocate for the development of a new collaboratively developed evidence-based tool rooted in qualitative methodology and best practice methods. The conclusion emphasizes the necessity of a tool that can provide a comprehensive judgement on the methodological limitations of a QES, addressing the needs of end-users, and ultimately enhancing the trustworthiness of QES findings in decision-making processes.

## Background

As qualitative evidence syntheses (QES) are becoming more common and increasingly used in decision-making processes [1–5], there is a need for a tool to assess the methodological limitations of a complete QES. This methodological assessment tool could help users to understand the trust they can place in the findings of a QES and help to interpret further use. In our work, this type of assessment tool would primarily be useful when an existing QES is found that answers a commissioner’s question. In this case, we need to be able to assess the methodological limitations of the completed QES to make a judgement for the commissioner on the extent to which the findings can be trusted and used to suit their purposes. We refer to an assessment tool, and not a checklist, as a deeper methodological understanding of the limitations of a QES is needed to assess the synthesis and how its methodological limitations impact on further use. We believe that the scoring or ranking which are the products of a checklist would not allow for a deep enough evaluation of and reflection around the methodological limitations of the QES and how they relate to the context and question that the QES is going to be used in and for.

The foundation for the discussion in this commentary was a teaching experience the team had in 2022. A request for course content was how to assess the methodological limitations of a QES. We recently had an in-house discussion about the three tools we had identified as options for assessing the methodological limitations of a QES. All three tools are in beta or preliminary versions. We discovered that the assessment tools are not easily accessible. Knowledge of their existence and whereabouts is necessary to locate them. All tools have been developed to meet an internal need (fit for purpose). None of them have been developed through best practice methods [6, 7]. The three tools are:

- Tool 1*: Criteria for assessing how well a qualitative evidence syntheses (systematic reviews of qualitative studies) was conducted*, a tool developed by Lewin and colleagues in 2012 [8, 9]
- Tool 2: a prototype assessment tool based on AMSTAR 2 [10], Measurement Appraisal Checklist to Assess Qualitative Evidence Syntheses (MACAQuES) by Booth and colleagues from 2019 [11]
- Tool 3: *Review template for qualitative evidence synthesis (QES)*, developed by the Swedish Agency for Health Technology Assessment and Assessment of Social Services (SBU) based on the ENTREQ reporting guidance [12] in 2023, is published but is still marked as “under development” [13]

We wanted to expand our in-house discussion further for teaching purposes. To do this, first, the authors compared the QES methodological assessment tools in a table and through discussion. Next, we incorporated them into an introductory course on QES methods delivered in October 2022. During the course, we had students reflect over any topics or questions they felt were missing from the existing tools based on the course content. Finally, we reflected on the student feedback and our experiences to assess and conclude that none of the tools fully met our needs.

In this short note, we aim to briefly present and compare items across the three assessment tools we identified and describe what we believe to be their strengths and limitations.

## Three assessment tools

We have compared the three QES assessment tools (see Table 1). An x was placed in the table if an item was mentioned in a question or a prompt.

**Table 1:** A comparison of the topics covered in the three assessment tools

**Topic**	**Tool 1** [8, 9]	**Tool 2** [11]	**Tool 3** [13]	**Total**
Planning / protocol		X	X	2
Patient etc involvement		X		1
Researchers’ competence			X	1
Review question	X	X	X	3
Inclusion criteria	X	X	X	3
Literature search	X	X	X	3
Screening			X	1
Description of excluded studies	X	X		2
Description of included studies		X		1
Methodological assessment of included studies	X	X	X	3
Data extraction		X		1
Synthesis	X	X	X	3
Findings	X	X	X	3
Dissemination bias		X		1
Reflexivity	X	X	X	3
Conflict of interest		X	X	2
Confidence in findings		X	X	2
Other			X	1
Total	8	15	13	

Seven of the eighteen topic areas are covered in all three tools (review question, inclusion criteria, literature search, methodological assessment of the included studies, analysis/synthesis, findings and reflexivity). Four of the topic areas are covered by two tools; a description of the excluded studies is covered in tool 1 and tool 2. Planning/protocol, conflict of interest, and confidence in the findings are covered in tool 2 and tool 3. However, tool 1 was published before the use of GRADE CERQual was implemented, so it is not surprising that that topic area is missing. Six topic areas are covered only by one assessment tool; tool 2 asks users to think through patient involvement, the description of the included studies, data extraction/coding, and dissemination bias. Tool 3 asks users to reflect on researchers’ competence, screening, and other.

Tool 1 has considerably fewer topic areas but includes prompt questions to help the user think through the topic areas. Tool 2 also provides prompt questions or items of note that users should consider when thinking through the topic area. Tool 3 is accompanied by a user guide.

All three tools require experience with and knowledge of qualitative research. This knowledge is needed to interpret the items/questions in a “qualitative manner” to ensure that methodological limitations relevant to qualitative research are assessed. Many questions are not explicitly formulated, meaning that the end user needs to understand qualitative research principles and practices to interpret and apply them. For example, a detailed knowledge around searching and where relevant qualitative evidence is located [13], a knowledge of which synthesis method is appropriate for which type of question [8, 9, 11, 13], and a knowledge of the QES authors background, experience, and competence [13]. Finally, the tools raise concepts that may be new to some researchers such as the concept of the impact of dissemination bias in primary qualitative research and its implications on QES findings [11].

## Need for collaboration in developing a new evidence-based methodological assessment tool for QES

Based on our comparison of the three assessment tools, we think there is a need to systematically search for map and assess existing tools. If there is not an existing tool which has been developed in an evidence-based way, then a tool should be considered. Ideally, the end goal would be to develop a new assessment tool that is based on the principles of *qualitative* research and qualitative evidence syntheses using best practice methods for assessment tool development. The development of the new tool should follow best practice methods so that it reflects all items relevant for the assessment of a completed QES, is based on qualitative methodology, and addresses the needs of the end user—being able to assess the limitations of a completed QES.

This process should be a collaborative effort within the QES community. The first step would be a systematic search for existing tools and the identification of relevant principles in these tools. Additional principles should be gathered from focus groups. This exploratory step would be followed by a Delphi process where stakeholders could come to an agreement on the principles that should be included in a future tool. After the consensus process has been completed, an assessment tool could begin to be developed and user tested.

Recently, this process of collaboratively developing an evidence-based tool for the assessment of the methodological limitations of primary qualitative studies included in a QES (CAMELOT) has been completed [5, 14–16]. The CAMELOT project followed the same process we describe above, involving a large number of relevant stakeholders in a collaborative process to determine what was important to include and how the tool could be used. CAMELOT, along with other previous [17–22] and ongoing [23] projects that have used the same methodology, lead us to believe that this process would lead to an evidence-based assessment tool for the assessment of the methodological limitations of a QES. We believe that the development of a tool for assessing the methodological limitations of a qualitative evidence synthesis is needed.

## Conclusion

In conclusion, we believe that none of the QES methodological assessment tools covered all of the areas that were raised by students as well as our reflections from working in the field. We found that the tools did not seem to be clearly grounded in qualitative research methods (for example words or expressions common in quantitative research were used). We also found that they could not provide a comprehensive/complete judgement on the methodological limitations of a QES that we could present to a commissioner or use to make a decision as critical areas or items were missing that we feel should be considered. We believe that the development of a tool for assessing the methodological limitations of a qualitative evidence synthesis is needed.

## Data Availability

Not applicable.

## References

[1] Lewin S, Glenton C, Lawrie TA, Downe S, Finlayson KW, Rosenbaum S (2019). Qualitative evidence synthesis (QES) for guidelines: paper 2–using qualitative evidence synthesis findings to inform evidence-to-decision frameworks and recommendations. Health Res Policy Syst.

[2] Downe S, Finlayson KW, Lawrie TA, Lewin SA, Glenton C, Rosenbaum S (2019). Qualitative evidence synthesis (QES) for guidelines: paper 1–using qualitative evidence synthesis to inform guideline scope and develop qualitative findings statements. Health Res Policy Syst.

[3] Glenton C, Lewin S, Lawrie TA, Barreix M, Downe S, Finlayson KW (2019). Qualitative evidence synthesis (QES) for guidelines: paper 3–using qualitative evidence syntheses to develop implementation considerations and inform implementation processes. Health Res Policy Syst.

[4] Flemming K, Noyes J (2021). Qualitative evidence synthesis: where are we at?. Int J Qual Methods.

[5] Munthe-Kaas HM, Glenton C, Booth A, Noyes J, Lewin S (2019). Systematic mapping of existing tools to appraise methodological strengths and limitations of qualitative research: first stage in the development of the CAMELOT tool. BMC Med Res Methodol.

[6] Moher D, Schulz KF, Simera I, Altman DG (2010). Guidance for developers of health research reporting guidelines. PLoS Med.

[7] Whiting P, Wolff R, Mallett S, Simera I, Savović J (2017). A proposed framework for developing quality assessment tools. Syst Rev.

[8] Lewin S (2018). Criteria for assessing how well a qualitative evidence syntheses (systematic reviews of qualitative studies) was conducted; EPOC resources for review authors.

[9] Lewin S, Bosch-Capblanch X, Oliver S, Akl EA, Vist GE, Lavis JN (2012). Guidance for evidence-informed policies about health systems: assessing how much confidence to place in the research evidence. PLoS Med.

[10] Shea BJ, Reeves BC, Wells G, Thuku M, Hamel C, Moran J (2017). AMSTAR 2: a critical appraisal tool for systematic reviews that include randomised or non-randomised studies of healthcare interventions, or both. BMJ.

[11] Booth AftCQaIMG (2019). Prototype - Measurement Appraisal Checklist to Assess Qualitative Evidence Syntheses (QES) (MACAQuES).

[12] Tong A, Flemming K, McInnes E, Oliver S, Craig J (2012). Enhancing transparency in reporting the synthesis of qualitative research: ENTREQ. BMC Med Res Methodol.

[13] SBU (2023). Granskningsmall för kvalitativa evidenssynteser (QES) (Tool to assess methodological limitations of qualitative evidence synthesis).

[14] Munthe-Kaas H, Bohren MA, Glenton C, Lewin S, Noyes J, Tunçalp Ö (2018). Applying GRADE-CERQual to qualitative evidence synthesis findings—paper 3: how to assess methodological limitations. Implement Sci.

[15] Munthe-Kaas HM, Sommer I, Noyes J, Cooper S, Garside R, Hannes K, et al. Development of the CAMELOT approach for considering methodological limitations of qualitative research in the context of GRADE-CERQual and qualitative evidence syntheses – protocol (version 1). Geneve: Zenodo; 2023.

[16] Munthe-Kaas AHB, Sommer I, Cooper S, Garside R, Hannes K, Noyes J. Developing CAMELOT for assessing methodological limitations of qualitative research for inclusion in qualitative evidence syntheses. Submitted to Cochrane Evidence Synthesis and Methods. 2024. 10.1002/cesm.12058 PMC11795985 40475879

[17] Page MJ, McKenzie JE, Bossuyt PM, Boutron I, Hoffmann TC, Mulrow CD (2020). The PRISMA 2020 statement: an updated guideline for reporting systematic reviews. BMJ.

[18] Page MJ, Moher D, Bossuyt PM, Boutron I, Hoffmann TC, Mulrow CD (2021). PRISMA 2020 explanation and elaboration: updated guidance and exemplars for reporting systematic reviews. BMJ.

[19] Page MJ, McKenzie JE, Bossuyt PM, Boutron I, Hoffmann TC, Mulrow CD (2021). Updating guidance for reporting systematic reviews: development of the PRISMA 2020 statement. J Clin Epidemiol.

[20] France E, Ring N, Noyes J, Maxwell M, Jepson R, Duncan E (2015). Protocol-developing meta-ethnography reporting guidelines (eMERGe). BMC Med Res Methodol.

[21] France EF, Cunningham M, Ring N, Uny I, Duncan EA, Jepson RG (2019). Improving reporting of meta-ethnography: the eMERGe reporting guidance. BMC Med Res Methodol.

[22] Cunningham M, France EF, Ring N, Uny I, Duncan EA, Roberts RJ (2019). Developing a reporting guideline to improve meta-ethnography in health research: the eMERGe mixed-methods study. Health Serv Deliv Res.

[23] Svendsen C, Whaley P, Vist GE, Husøy T, Beronius A, Di Consiglio E (2023). Protocol for designing INVITES-IN, a tool for assessing the internal validity of in vitro studies. Evid Based Toxicol.

